# Association Between Serum Amylin, Irisin, and Sirtuin 1 in Male Patients with Type 2 Diabetes and Diabetic Nephropathy

**DOI:** 10.5812/ijem-171383

**Published:** 2026-09-20

**Authors:** Sinhareeb Sabih Wajdi, Matin Mohyadini, Hasanain Mohamdhudifa Abdlwhab, Walaa E. Jassim, S. Zahra Bathaie

**Affiliations:** 1 Department of Clinical Biochemistry, Faculty of Medical Sciences, Tarbiat Modares University Tehran Iran; 2 Department of Salah Aldeen Health, Ministry of Health, Balad General Hospital Balad Iraq; 3 Department of Medical Laboratory Techniques, Faculty of Health and Medical Technologies, Central Medical University Baghdad Iraq

**Keywords:** Amylin, SIRT1, Irisin, Biomarkers, Diabetes, Diabetic Nephropathy

## Abstract

**Background:**

Type 2 diabetes mellitus (DM) is a progressive disease worldwide that leads to various complications. Given the roles of the hormones amylin and irisin and the enzyme sirtuin 1 (SIRT1) in metabolism and oxidative stress, we focused on these parameters in the serum of patients.

**Objectives:**

We investigated the associations between serum amylin, irisin, and SIRT1 levels and diabetic nephropathy (DN), an important complication of DM.

**Methods:**

Patients with DM (n = 50) and DN (n = 50) were included in this single-center cross-sectional study. Various parameters were measured from fasting blood and urine samples. Serum amylin, irisin, and SIRT1 levels were measured using enzyme-linked immunosorbent assay kits. In addition to demographic characteristics, fasting blood sugar, glycated hemoglobin, serum insulin, urine creatinine, and estimated glomerular filtration rate were used to classify participants.

**Results:**

After adjustment for age, diabetes duration, body mass index, fasting blood sugar, glycated hemoglobin, homeostasis model assessment of insulin resistance, triglycerides, low-density lipoprotein cholesterol, and high-density lipoprotein cholesterol, amylin levels were significantly lower, whereas irisin and SIRT1 levels were significantly higher, in the DN group than in the DM group. Multivariable logistic regression showed that higher amylin levels were associated with lower odds of DN, whereas higher irisin and SIRT1 levels were associated with higher odds of DN. Receiver operating characteristic curves were used to evaluate the ability of these biomarkers to discriminate between the DM and DN groups. In addition, mediation analysis showed associations among irisin, SIRT1, and DN.

**Conclusions:**

These findings suggest that irisin and SIRT1 are associated with DN status in patients with diabetes and may provide insights into metabolic complications. However, longitudinal studies are needed to establish causality.

## 1. Background

Diabetes mellitus (DM) is a group of metabolic disorders characterized by chronically elevated blood glucose levels due to impaired or insufficient insulin production, varying degrees of insulin resistance, or a combination of both (1, 2).

Insulin is the principal anabolic hormone, produced mainly by pancreatic beta cells. It consists of 2 peptide chains linked by disulfide bonds and stimulates glucose uptake from the circulation by adipocytes and myocytes (3). It regulates carbohydrate, fat, and protein metabolism. Insulin resistance occurs when cells become less responsive or unresponsive to circulating insulin. Consequently, some tissues, such as skeletal muscle, cannot respond adequately to insulin, preventing glucose from being used as an energy source. Blood glucose levels increase over time, and the pancreas compensates by producing more insulin. Insulin resistance is usually associated with DM, obesity, hypertension, and hyperlipidemia (4, 5).

Diabetic nephropathy (DN), a major complication of DM, is a chronic kidney disease that may lead to end-stage renal failure. Chronic hyperglycemia not only damages renal blood vessels but may also result in hypertension, proteinuria, and edema. Urinalysis and blood tests are used for diagnosis, while medications and lifestyle changes are among the treatment options. Early detection and treatment improve prognosis and quality of life (6, 7). In addition to insulin, other hormones and enzymes are altered in DM.

Amylin, also known as islet amyloid polypeptide (IAPP), is a 37-amino-acid neuroendocrine peptide hormone produced and released by pancreatic beta cells in parallel with insulin. Amylin cosecretion with insulin contributes to glycemic control through different mechanisms (8). It has been reported that amylin controls blood glucose by decreasing rapid insulin secretion and increasing basal insulin release (9, 10). In addition, amylin misfolding leads to aggregate formation. These aggregates disrupt cellular homeostasis in the pancreas of patients with DM and in kidney cells, contributing to renal failure (11).

Irisin, also known as a myokine, is a 112-amino-acid hormone-like polypeptide released from skeletal muscle in response to exercise (12). Irisin is generated when the extracellular carboxy-terminal portion of membrane-bound fibronectin type III domain-containing protein 5 (FNDC5) is cleaved and released. It is also highly expressed in other tissues, including the pancreas, heart, liver, adipose tissue, and brain. Irisin increases energy expenditure by stimulating the browning of white adipose tissue. In addition, as an islet hormone, it exerts a local vascular effect by reducing islet blood flow without affecting insulin secretion per se (13) and inhibits pyroptosis in beta cells of patients with DM (14). In contrast, some reports have shown that irisin enhances glucose-stimulated insulin secretion, improves PI3K/AKT insulin signaling, and inhibits inflammatory signaling in both in vitro and in vivo diabetes models (15). Furthermore, numerous studies have shown a close association between irisin and DM and metabolic diseases (12).

The sirtuin (SIRT) family comprises 7 conserved class III histone deacetylases. Nicotinamide adenine dinucleotide (NAD)-binding catalytic domains allow these proteins to target diverse substrates according to their biological functions. Sirtuins regulate glucose and lipid homeostasis, insulin production and sensitivity, fat mobilization, and oxidative stress (16, 17). Fewer mitochondria and, therefore, a marked reduction in oxidative phosphorylation have been reported in the muscles of patients with DM. Amylin misfolding due to overproduction in diabetes produces toxic oligomers and amyloid plaques within pancreatic islets, inducing beta-cell apoptosis and decreasing insulin secretion (18, 19). Loss of insulin secretion and ensuing chronic hyperglycemia result in systemic glucotoxicity and inflammation (20). Hyperglycemia and proinflammatory cytokines downregulate FNDC5 expression, the precursor of irisin, in muscle and adipose tissue. SIRT1 also influences FNDC5 expression through the AMP-activated protein kinase (AMPK) pathway. To our knowledge, no previous study has directly explored the relationship among amylin, irisin, and SIRT1 in patients with both DM and DN.

Although amylin, irisin, and SIRT1 have distinct sites of synthesis (pancreatic beta cells for amylin, skeletal muscle for irisin, and ubiquitous tissues for SIRT1), they converge on common pathophysiological pathways implicated in diabetic complications, including insulin resistance. Conflicting findings have also been reported regarding changes in their serum levels in patients with DM and DN.

## 2. Objectives

This study aimed to determine serum amylin, irisin, and SIRT1 levels in middle-aged male patients with DM and to evaluate their associations, particularly in those with DN.

## 3. Methods

### 3.1. Study Design and Setting

This cross-sectional study included 2 groups of male participants: 50 with DM and 50 with DN, with similar body mass index (BMI) distributions. Samples were collected from March 2024 to January 2025. Sample size and power calculations were performed using G*Power (Düsseldorf University, Germany). Based on preliminary data and previous studies of similar biomarkers in diabetic nephropathy, an effect size of Cohen d = 0.8 was assumed for the primary outcome, defined as the difference in serum biomarker levels (amylin, irisin, and SIRT1) between the DM and DN groups. A total sample size of 97 participants was calculated with a type I error (alpha) of 0.05 and statistical power (1 - beta) of 0.95. Ultimately, 100 patients were included. Participants were recruited at the Al Sadiq Pathology Laboratory in Balad District, Salah Aldeen Governorate, during routine blood and urine testing. All enrolled participants completed the study; there was no attrition.

### 3.2. Participants and Sampling

The inclusion criteria were male sex, age 40 - 65 years, and treatment with metformin and/or dipeptidyl peptidase-4 inhibitors for patients with DM. All participants had normal liver and heart function, protein status, and hematological profiles. The DN group comprised patients with DM and stage 2 or 3 chronic kidney disease according to the KDIGO 2024 classification (21). Diabetic nephropathy was diagnosed based on persistent albuminuria (urine albumin-to-creatinine ratio [UACR] ≥ 30 mg/g) and/or reduced estimated glomerular filtration rate (eGFR < 60 mL/min/1.73 m^2^) for at least 3 months, as confirmed by an endocrinologist using clinical history and laboratory findings. Patients with discordant renal findings (e.g., eGFR < 60 but UACR < 30, or UACR ≥ 30 but eGFR ≥ 60) were included only if their clinical history and imaging findings supported a diabetic origin of kidney disease and excluded other causes of nephropathy (e.g., primary glomerulonephritis or hypertensive nephrosclerosis). All diagnoses were made by a single endocrinologist (Dr. M. A. Hasanain) based on a comprehensive clinical evaluation.

The exclusion criteria were type 1 diabetes, cancer, age younger than 40 years or older than 65 years, female sex, insulin treatment, multiple complications, including cardiovascular disease and coronary artery disease, and stage 1, 4, or 5 kidney disease.

### 3.3. Data Collection and Instruments

After an overnight fast of 10 - 12 hours, venous blood samples were collected, and serum and plasma were separated. Enzymatic assays were used to measure fasting blood sugar (FBS) by the glucose oxidase/peroxidase method (LOT No. 11503); total cholesterol (TC) by the cholesterol oxidase/peroxidase method (LOT No. 11805); triglycerides (TG) by the glycerol phosphate oxidase/peroxidase method (LOT No. 11828); high-density lipoprotein cholesterol (HDL-C) by the phosphotungstate/Mg-cholesterol oxidase/peroxidase method (LOT No. 11523); urea using urease/salicylate (LOT No. 11536); and creatinine by the Jaffe method (LOT No. 11802), using an Auto Spectrophotometer Biosystem (Barcelona, Spain). Low-density lipoprotein cholesterol (LDL-C) was estimated using the formula -->FORMULA<-- (22). Insulin was measured using a chemiluminescent immunoassay (Biosystem, Barcelona, Spain).

Insulin resistance was estimated using the homeostasis model assessment of insulin resistance (HOMA-IR), calculated as -->FORMULA<--.

Glycated hemoglobin (HbA1c) was assessed using the CLOVER A1c Plus Analyzer (OSANG Healthcare, South Korea). Microalbumin was quantified in the first morning urine sample using an appropriate kit (Boditech Med Co., South Korea) according to the manufacturer’s instructions. Amylin, irisin, and SIRT1 levels were measured using enzyme-linked immunosorbent assay kits at the International Research Center (Dr. Ali Abdul Hussein) in Baghdad, Iraq. The following kits were used: Human Serum Amylin/DAP, Cat. No. ABIN6968062 (Limerick, PA, USA), with a sensitivity of 9.375 pg/mL, detection range of 15.6 - 1000 pg/mL, intra-assay coefficient of variation (CV) < 8%, and inter-assay CV < 10%; Human Irisin, Cat. No. E-EL-H6120 (Elabscience Bionovation Inc., Houston, TX, USA), with a sensitivity of 9.38 pg/mL, detection range of 15.63 - 1000 pg/mL, and intra- and inter-assay CVs < 10%; Human SIRT1, Cat. No. E-EL-H1546 (Elabscience Bionovation Inc., Houston, TX, USA), with a sensitivity of 0.19 ng/mL, detection range of 0.31 - 20 ng/mL, and intra- and inter-assay CVs < 10%; and Creatinine Assay Kit, Cat. No. M11502p-23 (Biosystem, Barcelona, Spain), with a limit of detection of 0.1 mg/dL creatinine (= 8.84 µmol/L creatinine) and a limit of linearity of 20 mg/dL (= 1768 µmol/L creatinine).

The eGFR was calculated from serum creatinine, age, weight, and height using the CKD-EPI equation (23). BMI was calculated as weight in kilograms divided by height in meters squared.

### 3.4. Statistical Analysis

Group comparisons between patients with DM and DN were performed using independent t tests for normally distributed variables, presented as mean ± standard deviation (SD), and Mann-Whitney U tests for skewed data, presented as median (Q1 - Q3). Multivariable logistic regression was used to assess the associations of amylin, irisin, and SIRT1 with DN, adjusting for age, diabetes duration, BMI, FBS, HbA1c, HOMA-IR, TG, LDL-C, and HDL-C. Receiver operating characteristic (ROC) curves were used to determine discriminatory accuracy for DN versus DM, with area under the curve (AUC), sensitivity, and specificity reported. Nonlinear associations between amylin, irisin, and SIRT1 and DN were examined using restricted cubic splines (RCS) with 4 knots placed at the 5th, 35th, 65th, and 95th percentiles for each parameter, with the median of each parameter as the reference. All other analyses were performed using Python (RRID: SCR_024202_, version 3.12) with NumPy (RRID: SCR_008633_, version 1.26), Pandas (version 2.1.4), Matplotlib (RRID: SCR_008624_, version 3.8.1), Scikit-learn (RRID: SCR_002577_, version 1.4.0), and SciPy (RRID: SCR_008058_, version 1.13.1), and R (version 4.5.1) with the "mediation" package to explore potential direct and indirect associations between SIRT1 and DN via irisin. Given the cross-sectional design, mediation results were interpreted as exploratory and consistent with a mediating role, but causal inference could not be established (24, 25). Differences were considered significant at P < 0.05.

### 3.5. Ethical Considerations

The study was conducted in accordance with the Declaration of Helsinki, and all procedures involving human participants were approved by the Ethical Committee of Tarbiat Modares University (Approval Code: IR.MODARES.REC.1403.098). All participants provided written informed consent.

## 4. Results

### 4.1. Baseline Characteristics

Significant differences were observed in several baseline characteristics between the DN and DM groups (Table 1). The DN group was older (P < 0.001) and had a longer duration of diabetes (P = 0.001). Serum levels of key biomarkers also differed significantly: amylin was lower in the DN group (P = 0.002), whereas irisin (P < 0.001) and SIRT1 (P < 0.001) were higher than those of the DM group.

**Table 1.** Baseline Characteristics of All Patients ^a^

Variables	DN	DM	P-Value
**Age (y)**	59.0 ± 6.5	53.4 ± 6.2	< 0.001
**Diabetes duration (y)**	12.5 (8 - 18)	8.5 (6 - 12.25)	0.001
**BMI (kg/m^2^), continuous**	30.1 ± 5.3	29.9 ± 4.6	0.853
**BMI classification**			0.627
Normal (18.5 - 24.9)	7 (7.0)	4 (8.0)	
Overweight (25 - 29.9)	23 (46.0)	24 (48.0)	
Obese (≥ 30)	20 (40.0)	22 (44.0)	
**FBS (mg/dL)**	142.0 ± 48.9	164.3 ± 65.5	0.057
**HbA1c (%)**	8.02 ± 2.00	8.42 ± 1.50	0.267
**Insulin (µIU/mL)**	16.2 (9.6 - 23.2)	13.4 (9.6 - 21.0)	0.605
**HOMA-IR**	5.22 (3.51 - 7.63)	4.85 (3.67 - 9.39)	0.793
**Serum urea (mg/dL)**	45.7 ± 15.4	31.8 ± 7.1	< 0.001
**Serum creatinine (mg/dL)**	1.38 ± 0.22	0.92 ± 0.08	< 0.001
**eGFR (mL/min/1.73 m^2^)**	61.1 ± 10.1	100.1 ± 8.3	< 0.001
**Urine albumin (mg/L)**	13 (10.75 - 28.5)	10 (8 - 10.25)	< 0.001
**TG (mg/dL)**	133 (87.75 - 175)	134 (82.75 - 184.25)	0.684
**Total cholesterol (mg/dL)**	173.5 ± 44.7	197.1 ± 42.1	0.008
**LDL-C (mg/dL)**	102.3 ± 42.6	123.0 ± 41.3	0.015
**HDL-C (mg/dL)**	42.4 ± 9.0	42.7 ± 9.0	0.890
**Amylin (pg/mL)**	101.0 ± 16.4	112.2 ± 17.9	0.002
**Irisin (pg/mL)**	384.0 (307.1 - 436.0)	184.5 (142.0 - 254.6)	< 0.001
**SIRT1 (ng/mL)**	8.28 ± 1.86	6.20 ± 2.32	< 0.001

^a^ Values are expressed as mean ± SD, median (Q1 - Q3), or n (%). Abbreviations: BMI, Body Mass Index; FBS, fasting blood sugar; HbA1c, glycated hemoglobin; HOMA-IR, homeostatic model assessment of insulin resistance; TG, triglycerides; LDL-C, low-density lipoprotein cholesterol; HDL-C, high-density lipoprotein cholesterol; eGFR, estimated glomerular filtration rate.

### 4.2. Kidney Function Parameters

Table 1 shows that the DN group had significantly impaired kidney function compared with the DM group. Serum urea and creatinine levels were higher in the DN group (both P < 0.001). The eGFR was significantly lower in the DN group (P < 0.001), reflecting impaired glomerular filtration. Urine albumin levels were also significantly higher in the DN group (P < 0.001), indicating increased albuminuria and renal damage consistent with diabetic nephropathy.

### 4.3. Lipid Profile Parameters

Analysis of the lipid profile revealed differences between the DN and DM groups. Total cholesterol levels were significantly lower in the DN group (P = 0.008), as were LDL-C levels (P = 0.015). However, no significant differences were observed in TG or HDL-C (Table 1).

### 4.4. Multivariable Logistic Regression Analysis

Multivariable logistic regression was performed to assess the associations of amylin, irisin, and SIRT1 with DN (Table 2), adjusting for age, diabetes duration, BMI, FBS, HbA1c, HOMA-IR, TG, LDL-C, and HDL-C. Each unit increase in amylin was associated with a 5% reduction in the odds of DN (adjusted OR = 0.95, 95% CI: 0.92 - 0.98), with the third tertile showing a pronounced inverse association (adjusted OR = 0.11, 95% CI: 0.03 - 0.41) compared with the first tertile. In contrast, irisin was positively associated with DN, with each unit increase raising the odds by 1% (adjusted OR = 1.01, 95% CI: 1.01 - 1.02), and the third tertile markedly increased the odds (adjusted OR = 83.53, 95% CI: 11.73 - 594.61) compared with the first tertile. Similarly, SIRT1 was positively associated with DN, with each unit increase increasing the odds by 57% (adjusted OR = 1.57, 95% CI: 1.19 - 2.05), and the third tertile substantially increasing the odds (adjusted OR = 26.41, 95% CI: 5.51 - 126.47) compared with the first tertile.

**Table 2.** Multivariable Logistic Regression ^a^

Variables	Unadjusted OR	Adjusted OR ^b^
**Amylin (pg/mL), continuous**	0.96 (0.94 - 0.99)	0.95 (0.92 - 0.98)
T1	Ref	Ref
T2	0.42 (0.15 - 1.15)	0.35 (0.09 - 1.35)
T3	0.17 (0.06 - 0.49)	0.11 (0.03 - 0.41)
**Irisin (pg/mL), continuous**	1.01 (1.01 - 1.02)	1.01 (1.01 - 1.02)
T1	Ref	Ref
T2	3.15 (1.03 - 9.63)	2.08 (0.53 - 8.19)
T3	45.00 (10.24 - 197.73)	83.53 (11.73 - 594.61)
**SIRT1 (ng/mL), continuous**	1.67 (1.31 - 2.14)	1.57 (1.19 - 2.05)
T1	Ref	Ref
T2	2.64 (0.89 - 7.85)	3.11 (0.86 - 11.21)
T3	28.93 (7.62 - 109.82)	26.41 (5.51 - 126.47)

^a^ Values are expressed as (95% CI).

^b^ Adjusted for age, diabetes duration, body mass index, fasting blood sugar, HbA1c, HOMA-IR, TG, LDL-C, and HDL-C. T1, T2, and T3 represent tertiles 1 to 3. Outcome variable: DN = 1, DM = 0 (reference). OR > 1 indicates increased odds of DN.

### 4.5. Receiver Operating Characteristic Curve Analysis

Figure 1 and Table 3 present the ROC curve analysis evaluating the ability of amylin, irisin, and SIRT1 to discriminate between DN and DM, adjusted for the same confounders. Irisin had an AUC of 0.915 (P < 0.001), with 81% sensitivity and 93% specificity. SIRT1 had an AUC of 0.862 (P < 0.001), with 94% sensitivity and 71% specificity. Amylin had an AUC of 0.826 (P = 0.002), with 69% sensitivity and 93% specificity. These AUC values reflect discriminatory performance within this single dataset; external validation is needed to confirm generalizability.

**Table 3.** Receiver Operating Characteristic Curve Analysis ^a^

Variables	AUC ^b^	Cutoff	Sensitivity	Specificity	P-Value
**Amylin (pg/mL)**	0.826 (0.647 - 0.952)	105.05	69	93	0.002
**Irisin (pg/mL)**	0.915 (0.787 - 0.998)	385.96	81	93	< 0.001
**SIRT1 (ng/mL)**	0.862 (0.698 - 0.978)	4.88	94	71	< 0.001

^a^ Values are expressed as (95% CI) or percentage.

^b^ Area under the curve (AUC) after adjustment for age, diabetes duration, body mass index, fasting blood sugar, HbA1c, HOMA-IR, TG, LDL-C, and HDL-C.

**Figure 1.**
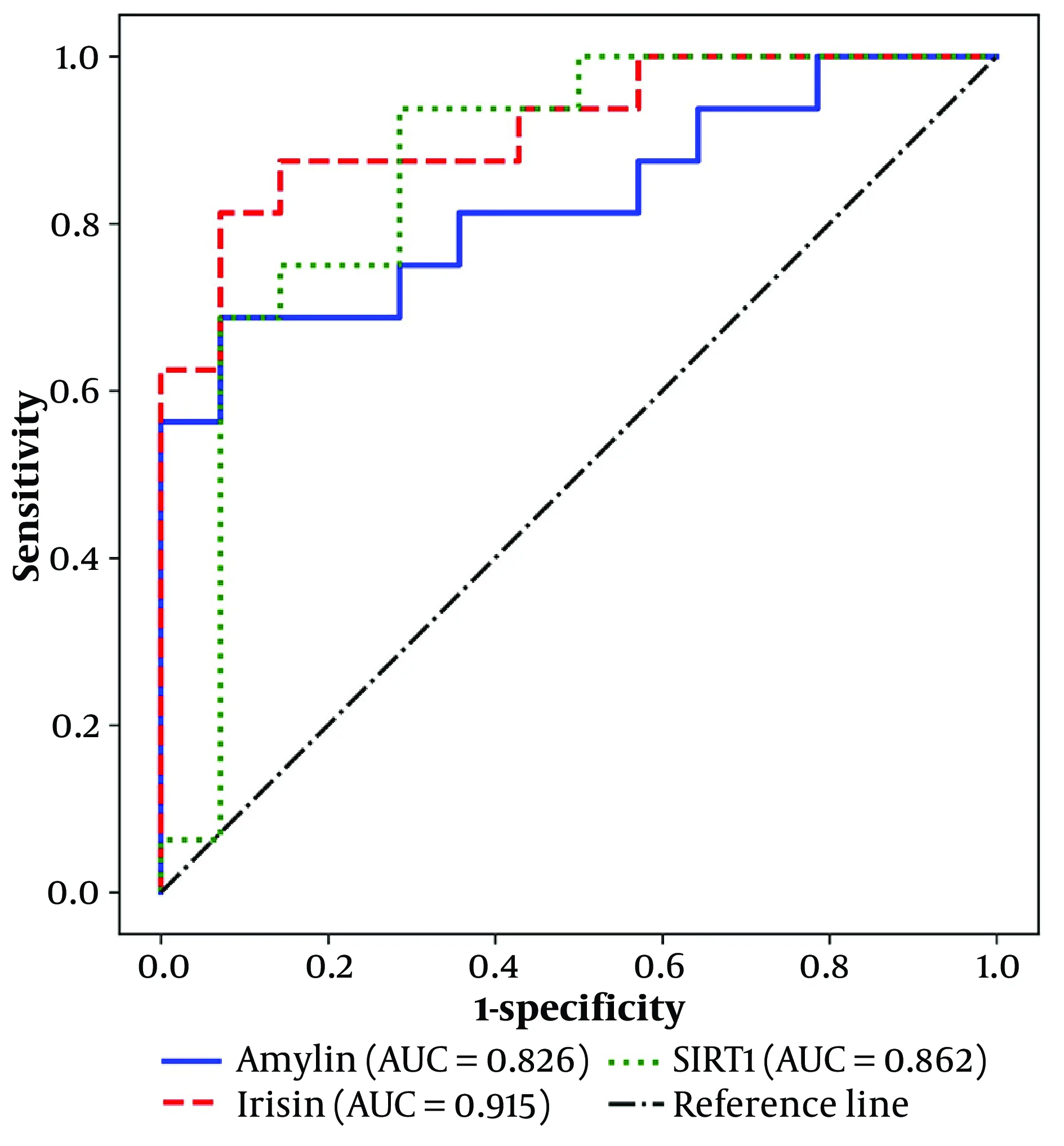
Receiver operating characteristic (ROC) curves. Sensitivity and specificity of amylin, SIRT1, and irisin for discriminating between the DM and DN groups.

### 4.6. Restricted Cubic Spline Analysis of Amylin, Irisin, and SIRT1

Figure 2 shows the results of restricted cubic spline (RCS) analysis exploring nonlinear associations between biomarker levels and the odds of DN. For amylin, Figure 2A shows a nonlinear association, with the odds of DN increasing significantly at levels below and above the reference threshold, reaching ORs of 2.0 at 80 pg/mL and 3.0 at 150 pg/mL, respectively (P for nonlinearity < 0.05), suggesting higher odds at both low and high concentrations. In contrast, SIRT1 exhibited a complex nonlinear pattern, with the odds of DN peaking at an OR of 5.0 at 8 ng/mL, followed by a decline at higher levels (P for nonlinearity < 0.05), indicating a potential threshold effect in which intermediate SIRT1 levels were associated with the greatest odds (Figure 2B). For irisin, the odds of DN increased with higher concentrations, peaking at an OR of 4.0 around 400 pg/mL before declining at higher levels (P < 0.05 for nonlinearity), suggesting a possible protective effect at elevated irisin concentrations (Figure 2C). These nonlinear associations demonstrate the relationships between these markers and the odds of DN, with patterns consistent across adjusted models. The reference points were the median of each parameter.

**Figure 2.**
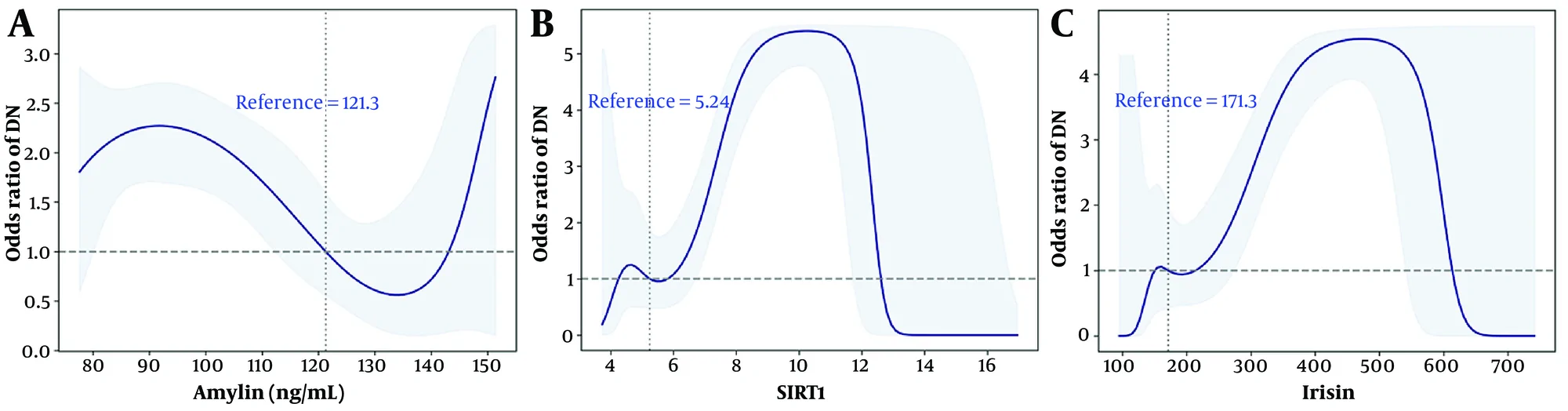
Restricted cubic spline (RCS) analysis. Association between serum amylin (A), SIRT1 (B), and irisin (C) concentrations and the odds of diabetic nephropathy. The model used 4 knots at the 5th, 35th, 65th, and 95th percentiles (reference: median). Solid lines indicate ORs; shaded areas indicate 95% CIs. Adjusted for age, diabetes duration, BMI, FBS, HbA1c, HOMA-IR, TG, LDL-C, and HDL-C. Abbreviations: OR, odds ratio; CI, confidence interval.

### 4.7. Mediation Analysis

The potential mediating role of irisin in the association between SIRT1 and DN was explored using path analysis (Figure 3). Given the cross-sectional design, these results are exploratory and do not imply causation. The indirect effect via irisin was statistically significant (coefficient = 0.0159, P < 0.001), whereas the direct effect of SIRT1 on DN independent of irisin was not significant (P = 0.216). Irisin accounted for 66.3% of the total association between SIRT1 and DN (P < 0.0001). Other potential mediation pathways involving amylin were not significant.

**Figure 3.**
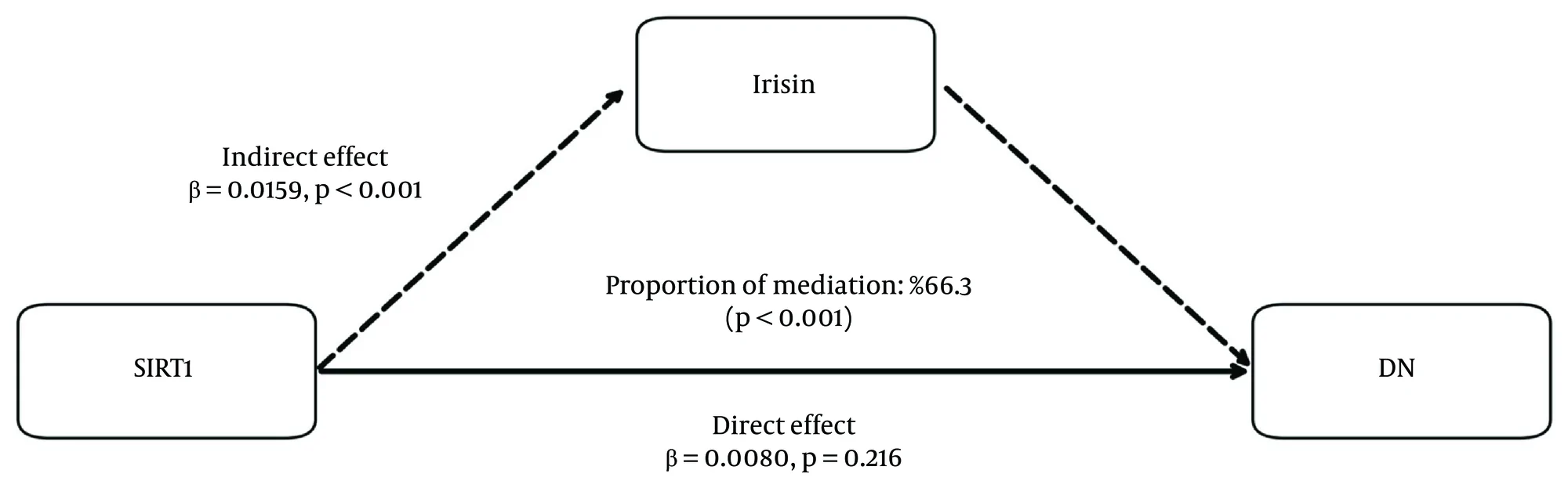
Exploratory mediation analysis. Direct and indirect associations between SIRT1, irisin, and DN.

## 5. Discussion

The association among amylin, irisin, and SIRT1 in DM and DN is of growing interest because of the potential of these molecules as biomarkers and therapeutic targets. Given controversies regarding serum amylin and irisin levels in patients with diabetes of different ages and sexes across studies, we investigated these parameters in middle-aged male patients with diabetes. Accordingly, 100 patients with DM were divided into 2 groups, with or without DN, based on clinical findings, and various anthropometric, biochemical, and hormonal parameters were evaluated.

Nonalbuminuric kidney disease caused by diabetes may be characterized by more advanced tubulointerstitial damage and vascular lesions, rather than the glomerular damage typically observed in albuminuric diabetes-related kidney disease (26). The prevalence of nonalbuminuric diabetic kidney disease is increasingly recognized, as studies show that a considerable number of patients with DM and reduced eGFR do not have albuminuria (27-29). In a Chinese cohort, 63.3% of patients with eGFR < 60 mL/min/1.73 m^2^ were normoalbuminuric (29). In an Indian cohort, nonalbuminuric diabetic kidney disease was identified in 39.1% of patients with reduced eGFR (28). According to our data, patients with DN showed a significant decrease in eGFR with increased albuminuria. Although there was a significant difference in proteinuria between the DM and DN groups, both remained within the normal range, reflecting normoalbuminuric diabetic kidney disease in some patients with DN. In this study, early-morning urine samples were collected as recommended by the kit manufacturer and as described in clinical biochemistry textbooks (30). However, 12- or 24-hour urine samples may be preferable for this test.

Chronic hyperlipidemia in diabetes results in microangiopathy, which contributes to renal damage and dysfunction. Microvascular damage is a critical factor in the development of DN (31, 32). Diabetic dyslipidemia exacerbates renal damage by inducing inflammation and oxidative stress, which are critical components in DN progression (31, 33-35). In our study, patients had stage 2 or 3 DN. Thus, lower levels of cholesterol, TG, and LDL-C in patients with DN may indicate impaired kidney function, as reflected by decreased eGFR due to damage to the glomerular filtration barrier, or may reflect the use of drugs such as statins and angiotensin-converting enzyme inhibitors that lower lipid levels (36). In addition, reduced hepatic capacity for lipoprotein production has been observed in uremia and chronic inflammation (37), which should be considered in future studies.

According to our findings, amylin levels were significantly lower in patients with DN than in those with DM. As previously reported, hyperamylinemia initially occurs in the early stages of DM, leading to amylin aggregation. Amylin aggregates induce endoplasmic reticulum stress and impair mitochondrial function in renal cells, resulting in cellular dysfunction and renal failure. The toxic aggregated form of amylin causes beta-cell damage, which in turn leads to hypoamylinemia in later stages of the disease. This transition illustrates the complex interplay between metabolic abnormalities and the clinical progression of DM, characterized by substantial insulin resistance and beta-cell dysfunction (11, 38). Furthermore, multivariable regression and RCS analyses indicated that lower amylin levels were associated with increased odds of diabetic nephropathy in patients with DM.

As reported in the Results, irisin levels were significantly higher in patients with DN than in those with DM. However, reports on serum irisin levels in patients with diabetes are conflicting. Some studies reported lower circulating irisin levels in patients with DM and DN than in controls (39), whereas others reported increased levels in patients with DM (40-42). It has been suggested that irisin levels may reflect the metabolic status of patients with metabolic disorders (43). Notably, children with type 1 DM often have higher irisin levels, which are associated with better glycemic control (44). Irisin may protect against metabolic stress in DM and indicate the risk of developing DN in patients with normoalbuminuria (40). We also demonstrated an association between irisin levels and the odds of diabetic nephropathy in patients with DM.

In patients with diabetes and metabolic syndrome, elevated irisin levels may serve as a compensatory mechanism against insulin resistance. The anti-inflammatory properties of irisin may help alleviate insulin resistance (45), while its role in energy expenditure, glucose tolerance, and insulin sensitivity suggests a compensatory response to increased adiposity in humans (46-48). In addition, various lipid-lowering (49) and glucose-lowering (41) medications have been shown to increase serum irisin levels, potentially aiding in the management of diabetes and its complications.

Our results indicated that SIRT1, an NAD^+^-dependent deacetylase, was significantly higher in patients with DN than in those with DM. SIRT1 is an important regulator of glucose and lipid metabolism, mitochondrial function, and inflammatory responses. Restoring SIRT1 levels in diabetic mice has improved beta-cell function by reducing proapoptotic ceramide production, thereby attenuating adverse effects on insulin release (50). Higher SIRT1 levels also improve insulin sensitivity by mitigating mitochondrial dysfunction (51). Furthermore, elevated SIRT1 levels are associated with increased antioxidant activity (52) and reduced oxidative stress and inflammation, key factors in the progression of DM and its complications (53). Our results also indicated that increased SIRT1 levels in DN were associated with higher odds of diabetic nephropathy. However, the wide confidence intervals for the extreme tertiles indicate limited precision, likely due to the sample size, and these estimates should be interpreted cautiously.

Our exploratory mediation analysis revealed significant indirect associations between SIRT1 and DN through irisin, suggesting that irisin may mediate part of the relationship between SIRT1 and diabetic nephropathy. This statistical finding, together with previously reported biological links between these molecules (54-56), led us to propose a hypothetical pathway connecting SIRT1 and irisin. Briefly, irisin has been shown to stimulate SIRT1 via the AMPK-peroxisome proliferator-activated receptor gamma coactivator 1-alpha (PGC-1alpha) pathway, enhancing mitochondrial biogenesis and antioxidative capacity (54). In the context of diabetic nephropathy, increased SIRT1 could elevate irisin levels, possibly through the AMPK pathway, providing a potential mechanism for its protective effects. SIRT1 deacetylates and activates serine-threonine liver kinase B1 (LKB1), an upstream kinase that phosphorylates and activates AMPK (56). Once activated, AMPK enhances the expression of PGC-1alpha, a critical transcriptional coactivator for energy metabolism. PGC-1alpha, in turn, induces the expression of FNDC5, the precursor of irisin (54). This sequential pathway (SIRT1 → LKB1 → AMPK → PGC-1alpha → FNDC5 → irisin) suggests that elevating SIRT1 could increase irisin production, potentially reducing oxidative stress and inflammation in DN (55). However, our study measured only serum SIRT1 and irisin; intermediate molecules (LKB1, AMPK, PGC-1alpha, and FNDC5) were not assessed. Therefore, this pathway remains a hypothesis based on the literature and requires direct experimental validation in future research.

### 5.1. Study Limitations

The key limitations of the present study include the small sample size; therefore, future research should include larger samples.

Although age and diabetes duration differed significantly between the DM and DN groups, this was expected because DN is a late complication of diabetes that typically develops after long-standing hyperglycemia. These variables were adjusted for in all multivariable regression models to minimize confounding. We did not match or restrict these variables a priori because doing so would have substantially reduced the sample size. Nevertheless, residual confounding cannot be entirely excluded, and future studies with larger samples should consider matching or propensity score methods to further address this issue.

The cross-sectional design precludes conclusions regarding temporal or causal relationships. The ROC analyses were derived and tested in the same dataset, and no internal validation (e.g., bootstrapping) was performed. These parameters should be investigated in cohort studies that follow patients with DM for the possible development of DN.

To eliminate potential confounding effects of hormonal fluctuations associated with the female menstrual cycle, we included only male patients. However, this restricts the generalizability of our findings to female patients with type 2 diabetes. Future studies should include both sexes to determine whether similar associations exist in women.

Because the proposed signaling pathway discussed above is based on the literature, future studies should examine the molecular markers and proteins involved in this pathway. Similar studies could also be conducted in animal models to explore this pathway.

### 5.2. Conclusions

This study examined the associations of amylin, irisin, and SIRT1 in 2 groups of patients with diabetes, with or without DN. Irisin and SIRT1 are synthesized and secreted by distinct tissues; however, their potential interplay, as suggested by our mediation analysis and previous literature, warrants further investigation in mechanistic studies. As indicated by the ROC curves in this study, irisin and SIRT1 demonstrated discriminatory ability in this dataset. However, external validation is required before any clinical application. In addition to hyperglycemia and HbA1c, elevated irisin and SIRT1 levels may be associated with DN status. However, the cross-sectional design and lack of external validation preclude claims of clinical prediction or causality.

## Data Availability

The dataset presented in the study is available on request from the corresponding author during submission or after publication.
